# The Late King and the Hospitals

**Published:** 1911-04-15

**Authors:** 


### THE LATE KING AND THE HOSPITALS.

**Note—We shall be glad to receive information regarding further memorials in which alterations to existing institutions or the establishment of new hospitals are contemplated, in order to amplify this provisional list. Details about the estimates of cost and the nature of the schemes adopted will be acceptable.—Editors, "The Hospital."**

A COMMITTEE, representative of the four Provinces of the Union, has been formed, under the presidency of Lady. Gladstone, says the *Times* correspondent at the Cape, to consider the establishment of a memorial to King Edward, which will probably take the form of a hospital.

**Boston, Lincs.**—The Boston Hospital, it is announced, will be extended as the town's memorial to King Edward.

**Bournemouth.**—The town has started a fund to be divided between the Royal Boscombe and West Hants Hospital and the Royal Victoria Hospital, Bournemouth. It is now proposed that these institutions should be amalgamated.

**Bristol.**—A King Edward Memorial Infirmary is to be built near the Bristol Royal Infirmary. It will contain 180 beds, and three operating theatres.

**Bristol.**—A new wing, which will include a maternity ward, is to be built at the Bristol General Hospital as a memorial to King Edward VII. The wing is expected to cost £45,000.

**Buckinghamshire.**—The county memorial is to be a permanent trust fund for the relief of the sick and for the prevention of disease. Grants will be made to hospitals and sanatoria for open-air shelters and for the medical treatment of children.

**Cambridge** is raising £15,000 for a new out-patient department and a new children's ward as its memorial at Addenbrooke's Hospital.

**Cardiff.**—The payment of the debt on Cardiff Infirmary is the local memorial to the late King, which for the whole of Wales is to include a campaign against consumption.

**Colne (Lancs).**—Sir W. P. Hartley has promised to double the accommodation of the Jubilee Cottage Hospital, Colne, on condition that the endowment fund shall be increased by an equal amount to that which the enlargement will cost. The present number of beds is twelve; the extension will be the local memorial.

**Coventry** has collected £7,000 for the extension of the Coventry and Warwickshire Hospital, to include a new wing with top floor kitchens, a nurses' dining-room, and an isolation block. These will be paid for out of the King Edward Memorial Fund.

**Devizes.**—A children's ward is to be added to the hospital, towards which £500 has been given by Mr. A. Grant-Meek, as the town's memorial.

**Dorset.**—Dorset will commemorate King Edward by means of the King Edward VII. Dorset County Memorial Fund, the money contributed to be invested in the names of trustees and to be applied for the relief of sickness in the county, and especially for measures for combating tuberculosis.

**Ealing.**—The King has commanded that the new hospital which is being erected at Ealing, at a cost of £20,000, shall be named the King Edward Memorial Hospital. (On May 16 the Duchess of Argyll will open a bazaar at the Victoria Hall, Ealing, in aid of the building fund).

**Eastleigh, Hants.**—It is proposed to build a cottage hospital as a memorial. A site has been offered by Mr. Fleming.

**Eastbourne.**—The Borough's memorial to King Edward will consist of a new hospital on a new site.

**Hereford.**—All schemes are hanging fire, but the most promising is "for the improvement of Herefordshire General Hospital."

**Lincoln.**—The county and city will commemorate the late King by adding a new ward for women to the County Hospital at Lincoln and providing a nurses' home. The scheme is estimated to cost about £10,000, of which sum of £3,600 was promised at the meeting.

**Macclesfield.**—It is suggested the renovation and extension of the Infirmary here be regarded as the town and districts memorial.

**Romford, Essex.**—A new ward at the Victoria Cottage Hospital will be the local memorial.

**Rutland.**—Lord Lonsdale has promised to give the site for the proposed cottage hospital, at Oakham, which is to be the county's memorial.

**Sevenoaks.**—A new hospital for Sevenoaks and district is to be built at a cost of £10,000 as a memorial to King Edward. Lord Stanhope's private appeal has produced £3,300.

**Tynemouth.**—Here the Borough memorial is to take the form of an extension of the operating theatre at the Victoria Jubilee Infirmary, North Shields. It is hoped to raise £500 for the purpose.

**Wigan** Infirmary has decided to build a new out-patient department as a memorial to King Edward VII.

**Woking.**—As a memorial to the late King Edward, Woking has decided to raise £3,000 for the erection and partial endowment of a children's ward at its cottage hospital—which was established to commemorate the reign of Queen Victoria.

**Worthing.**—Contrary to expectations (see THE HOSPITAL, March 4, page 669), it has been decided to build, by public subscription, a new out-patients' department for the Worthing Hospital as the town's memorial.

